# Sleep quality in COVID-19 recovered patients

**DOI:** 10.5935/1984-0063.20220037

**Published:** 2022

**Authors:** Laith Thamer Al-Ameri, Ekhlas Khalid Hameed, Bilal S. Maroof

**Affiliations:** University of Baghdad, Al-Kindy College of Medicine - Baghdad - Iraq.

**Keywords:** COVID-19, Sleep Disorders, Circadian Rhythm, Sleep Latency

## Abstract

**Objective:**

Novel coronavirus disease 2019 is known to have poor impacts on health with health systems facing serious challenges. Limited information is available on health issues for patients who have recovered from the disease. Our study aims to investigate the extent of sleep disorders in patients who have recovered from the coronavirus disease.

**Material and Methods:**

A casecontrol study with 256 patients who had recovered from coronavirus disease 2019 was carried out, with 491 patients enrolled as the control. All participants were 18 years or older, and sleep quality was assessed using the Pittsburgh sleep quality index. Furthermore, sleep latency and hours needed for sleep were calculated. Chi-square and t-test statistical methods were used to analyze the variables.

**Results:**

A total of 215 (84%) recovered patients were associated with poor sleep quality, with 384 patients (78%) in the control group. The PSQI values for recovered and control groups were 8.77±3.7591 and 8.139±3.068, respectively, with a significant *p*-value of 0.014. Average hours needed for sleep were 6.899±3.7869 and 6.44±1.477 for recovered and control patients, respectively, with a significant *p*-value of 0.01. The difference in sleep latency was non-significant (*p*=0.374), with 29.01±39.3702 and 33.520±38.208 minutes for recovered and control patients, respectively.

**Conclusion:**

Sleep disorders were more prevalent among patients who had recovered from COVID-19 than the control group.

## INTRODUCTION

The novel coronavirus disease 2019 (COVID-19) has become a pandemic with the number of cases growing globally. With the rapid spread of COVID-19, various health systems are facing serious challenges. These challenges, although mainly emerging from physical health implications, may also have profound impacts on sleep and mental health^1^.

Adequate sleep is essential to the survival of the human being. Sleep provides essential refreshing, protective, restorative, and energy-conserving functions. Many people around world have been faced with worries and fear about their safety, the lack of an effective treatment or vaccine, and unexpected socioeconomic consequences such as unemployment and difficulty of access to necessary commodities due to the pandemic^2,3^. Psychosocial stressors, such as fear, stress, anxiety, changed lifestyle, and inadequate social support may impact the sleep patterns among the population. Generally, sleep disturbances have been associated with adverse health consequences, including alteration to the immune system, metabolism, protein catabolism, and nitrogen balance^2,4^.

Sleep disruption affects cognitive skills and the quality of life; additionally, sleep disorders can decrease pain thresholds, and increase depression and anxiety^5^. These issues may have multiple impacts on mental health across populations, necessitating the attention of global health researchers and practitioners. Recognizing and treating sleep disorder is particularly vital during stressful times, such as the COVID-19 pandemic, as it may significantly reduce morbidity and mortality.

Many medical conditions have been shown to be related to poor sleep quality, such as following recovery, bacterial and viral meningitis, brain trauma, and even general anesthesia, which have a consequent impact on cognitive skills and mental health^6-8^.

To the best of our knowledge, there is a scarcity of research on the extent of sleep disorders among individuals who recovered from the illness. This article addresses sleep disorders among patients who have recovered from COVID-19.

## MATERIAL AND METHODS

This is a case-control study that was carried out from January 2021 to March 2021 with 747 participants, 256 of which had previously been diagnosed with COVID-19 with subsequently recovered, while 491 participants were enrolled as age- and sex- matched controls.

Patients selected for the first group (COVID-19 recovered patients) were those who had been previously diagnosed by RTPCR test according to protocols used in different nations with a subsequent recovery confirmed with two negative RT- PCR tests and up to three months as a recovery period. Of the first group, a subgroup of patients who need a respiratory care unit (RCU) admission were recorded and compared to recovered patients with no previous admission or who had been admitted to hospital wards with no need of respiratory assistance. Control participants were from the general population, with neither COVID-19-related symptoms, nor having been in contact with known COVID-19 patients.

Exclusion criteria for both groups were individuals with a still active infection, incapacitating condition, individuals with chronic treatment with hypnotics before the pandemic, or any medical conditions that may affect the quality of sleep, such as morbid obesity, traumatic brain injury, and others.

A questionnaire was distributed to all individuals using a Google Form application and distributed through social media using sponsored advertising, set to include all Arabic nations. The questionnaire was preceded with full clarification of the purpose of the study. Acceptance to enroll in the study and being 18 years of age or older were prerequisite. Questions were designed to include age, gender, and specific questions related to sleep quality scoring. Additional questions were added to the COVID-19 recovered group, including time since recovery and whether admission to the respiratory care unit was required.

Sleep quality was assessed through the self-reported Pittsburgh sleep quality index (PSQI) translated to the Arabic language^9^. PSQI has been validated to Arabic language just prior to participant enrolment at January 2021.

Data were collected and assembled with the Excel program to calculate PSQI scores. The global PSQI score was calculated through the summation of its seven components, with overall scores ranging from 0 to 27. The higher the score, the poorer the sleep quality of respondents. A score higher than five was regarded as poor quality.

Statistical analysis of data was carried out using the IBM SPSS 22 software for Windows (Chicago, IL, USA). Different variables were calculated through chi-square and t-test, where a *p*-value of <0.05 was considered significant. Shapiro-Wilk and Levene’s tests were used to check the normality of the distribution of dependent variables, as well as to determine the homogeneity of variance of the dependent variables.

Ethical approval was obtained from the ethical and scientific unit in Al-Kindy College of Medicine, University of Baghdad, after reviewing the study protocol.

## RESULTS

Among the 747 patients enrolled in the study, 325 were males. The male to female ratio was 1:1.3, the mean age for all enrolled individuals was 36.5±9.002 years, the mean age for COVID-19 recovered patients was 37.09±9.144 years, and the mean age was 36.19±8.921 years for the control group; see Table 1.

**Table 1:** Mean age with standard deviation for both groups.

Group	N	Mean age	Standard deviation	*p*-value	Test
COVID-19 recovered	256	37.09	9.144	0.195*	t-test
Control	491	36.19	8.921		

Note:

* *p*-value is not significant with 95%CI.

Sleep quality was assessed by calculation of global PSQI scores. The mean score was 8.77±3.7591 for COVID-19 recovered patients and 8.139±3.068 for controls, with a significant *p*-value of 0.014 when comparing means with t-test. Referring to the comparison sleep quality for both groups, according to numbers of individuals with poor sleep quality (PSQI score more than 5), 215 (84%) of COVID-19 recovered group and 384 (78%) of the controls were recorded as having poor sleep quality, with a significant *p*-value of 0.044 using the chi-square test; see Table 2.

**Table 2:** Sleep quality data for both groups.

	COVID-19 recovered	Control	*p-*value	Test value	df	Method
Sleep quality (PSQI) (scoring)**	8.77±3.7591	8.139±3.068	0.014*	2.463 t-value	745	t-test
Sleep quality (PSQI) (No. of patients)	215 (84%)	384 (78%)	0.044*	4.049 X^2^ value		chi-square
Sleep hours/day**	6.899±3.7869	6.44±1.477	0.01*	2.321 t-value	745	t-test
Sleep latency (min.)**	29.01±39.3702	33.520±38.208	0.374	-1.515 t-value	745	t-test

Notes:

* *p*-value is significant with 95%CI;

** mean ± standard deviation.

The average sleep hours needed per day was 6.899±3.7869 hours for COVID-19 recovered patients and 6.44±1.477 hours for the controls, with a significant *p*-value of 0.01.

Sleep latency was 29.01±39.3702 minutes for COVID-19 recovered patients and 33.520±38.208 minutes for the controls, with a non-significant *p*-value of 0.374; see Table 2.

Relating the sleep quality of COVID-19 recovered patients and their need to be admitted to an RCU, of 256 patients, 13 were admitted to an RCU, who had a mean PSQI of 8.687±3.764. In contrast, for the other patients with no need for RCU admission, the mean PSQI was 10.308±3.637, with a non-significant *p*-value of 0.767; see Table 3.

**Table 3:** Sleep quality (PSQI) in recovered patients with previous admission to respiratory care unit.

Group	N	PSQI*	*p*-value	t-value	df	Method
No RCU admission	243	8.687±3.764	0.767	-1.515	254	t-test
RCU admission	13	10.308±3.637	8.921		254	

Notes:

* *p*-value is significant with 95%CI;

** mean ± standard deviation.

## DISCUSSION

According to the above results, with a PSQI score more than five being considered as poor sleep quality, sleep disorders were more prevalent among patients who had recovered from COVID-19 than the control group, where the PSQI scores were 8.77±3.7591 and 8.139±3.068, respectively, with a significant *p*-value of 0.014. However, both were significant, compared to the general population before the announcement of COVID-19 as an epidemic^10^.

The high average PSQI score for the general population at the time of study could be due to psychological effects, such as stress, related to the outbreak. This stressful situation can activate the fight-or-flight mechanisms in response to danger, which may directly affect sleep quality. This is consistent with the study of Huang and Zhao (2020)^11^, who stated that people are spending too much time thinking about the outbreak and the life-threatening disease. According to Cellini et al. (2020)^12^, most people during the COVID-19 outbreak have felt anxiety and experienced psychological changes, including sleep disorders, during the lockdown in Italy.

The significantly higher PSQI score of patients who had recovered from COVID-19 is a newly emergent health issue, among many health issues following the disease that needs explanation. A possible explanation is related to the viral genome type of SARS-CoV-2, which is responsible for the disease. This virus is a double-stranded RNA virus, which initiates an acute phase response mediated by the antiviral cytokine interferon^13^. This response refers to the rapid and early activation of immune responses to infections and injury.

In a study by Waltuch et al. (2020)^14^, elevated cytokine levels in children with COVID-19 post-infection were demonstrated; additionally, Santiago et al. (2015)^15^ stated that most cytokines could stay elevated, even after the curing of viral infections. In another experimental study by Pandey and Kar (2011)^16^, it was found that cytokine levels (IL-1 p, IL-6, and IL-12) were elevated in rats with rapid eye movement (REM) deprivation. These elevations increased directly with the extent of sleep loss, as suggested by previous referred researches, the increase in cytokine levels were found to induce a poor sleep quality. Furthermore, even at a small dose and when given centrally or systemically, certain cytokines (mainly TNF or IL1) were found to have a sleep induction effect in both humans and animals with the non-rapid eye movement sleep (NREMS) is increased. As the dose is increased, more increase in NREMS occurs at the expense of decreased REMS. At high doses both NREMS and REMS are inhibited. Additionally, microinjection of TNF or IL1 into the hypothalamic or even near to it or other sleep regulatory circuits is shown to stimulate NREMS, increase sleepiness, in addition to enhancing (EEG) and power during NREMS^17^.

Another suggested explanation for the high prevalence of sleep disorders in COVID-19 recovered patients is related to their persistent symptoms after recovery. Yelin et al. (2020)^18^ have suggested that many patients show long-term consequences and complications after COVID-19 infection; however, studies are still limited concerning the long-term complications following COVID-19 infection.

In our study, the average sleep hours needed per day were 6.899±3.7869 hours for the COVID-19 recovered group and 6.44±1.477 hours for the controls, with a significant *p*-value of 0.01. The greater number of sleeping hours needed by COVID- 19 recovered patients could be due to the fatigue that the recovered patients suffered after the viral infection. This is in agreement with Wilson^19^, who stated that, after the SARS outbreak in 2002; many people who were infected reported experiencing fatigue and sleep disturbances.

Regarding sleep latency, we found that it was 29.01±39.3702 minutes for the COVID-19 recovered group and 33.520±38.208 minutes for the controls, with a non-s ignificant *p*-value of 0.374. However, studies illustrating the relationships between viral infections and the time needed for sleep onset remain limited. Also, as recovered COVID-19 infected patients presented higher PSQI score, it is expected that they are sleep restricted which diminishes the latency to sleep.

Some of the COVID-19 patients also reported associated RCU admission; these respondents showed higher PSQI scores (10.308±3.637), compared to COVID-19 patients with no history of RCU admission (8.687±3.764). However, this picture was non-significant, with a *p*-value of 0.767. Many other studies have presented significant findings for the effect of mechanical ventilation on sleep^20^. Our explanation was related to the small size of the sample in the RCU-admitted group, with respect to the non-RCU group (13 and 243 individuals, respectively); therefore, a future study is needed with more accurate sampling, in order to confirm the results obtained herein.

Our study had several limitations. For example, it was based on a PSQI questionnaire, which was filled out online and sleep laboratory tests were unavailable. We suggest future studies using well-equipped sleep laboratories, in order to demonstrate the long-term effect of COVID-19 on different sleep cycles. In addition, control group participants have not been tested to RT-PCR to confirm their not having the infection. Another important limitation was the unavailability of medication information used in management, as this was a multi-national study with different guidelines used for such management. Medication information is an important issue in order to assess the effects of such medications on sleep quality.

## CONCLUSION

Sleep disorders were more prevalent among patients who had recovered from COVID-19 than the control group, although both showed worse sleep quality than the general population before the announcement of the pandemic.
